# Left atrial minimal volume: association with diastolic dysfunction and heart failure in patients in sinus rhythm or atrial fibrillation with preserved ejection fraction

**DOI:** 10.1186/s12880-021-00606-3

**Published:** 2021-05-06

**Authors:** Assaf Ben-Arzi, Evgeni Hazanov, Diab Ghanim, Guy Rozen, Ibrahim Marai, Liza Grosman-Rimon, Erez Kachel, Offer Amir, Shemy Carasso

**Affiliations:** 1 Cardiovascular Institute, Poriya Medical Center, Lower Galilee, Israel; 2 The Azrieli Faculty of Medicine in the Galilee, Bar-Ilan University, Zefat, Israel; 3 Department of Cardiothoracic Surgery, Leviev Heart Center, Sheba Medical Center, Tel HaShomer, Israel; 4 Department of Cardiology, Hadassah Medical Center, Faculty of Medicine, Hebrew University of Jerusalem, Jerusalem, Israel

**Keywords:** Left atrial volumes, Diastolic dysfunction, Heart failure, Atrial fibrillation, Preserved ejection fraction

## Abstract

**Background:**

Evidence of diastolic dysfunction (DD) required for the diagnosis of heart failure with preserved ejection fraction (HFpEF) is elusive in atrial fibrillation (AF). Left ventricular (LV) and left atrial (LA) speckle-tracking echocardiography (STE) may provide rhythm independent indications of DD. We aimed to find common LV/LA myocardial mechanics parameters to demonstrate DD, using STE in patients with AF.

**Methods:**

176 echocardiographic assessments of patients were studied retrospectively by STE. 109 patients with history of AF were divided in three groups: sinus with normal diastolic function (n = 32, ND), sinus with DD (n = 35, DD) and patients with AF during echocardiography (n = 42). These assessments were compared to 67 normal controls. Demographic, clinical, echocardiographic and myocardial mechanic characteristics were obtained.

**Results:**

The patients with DD in sinus rhythm and patients with AF were similar in age, mostly women, and had cardiovascular risk factors as well as higher dyspnea prevalence compared to either controls or patients with ND. In the AF group, LV ejection fraction (LVEF) (*p* = 0.008), global longitudinal strain and LA emptying were lower (*p* < 0.001), whereas LA volumes were larger (*p* < 0.001) compared to the other groups. In a multivariable analysis of patients in sinus rhythm, LA minimal volume indexed to body surface area (Vmin-I) was found to be the single significant factor associated with DD (AUC 83%). In all study patients, Vmin-I correlated with dyspnea (AUC 80%) and pulmonary hypertension (AUC 90%).

**Conclusions:**

Vmin-I may be used to identify DD and assist in the diagnosis of HFpEF in patients with AF.

## Background

More than half of all patients suffering from heart failure (HF) have preserved left ventricle ejection fraction (LVEF) [1–6]. The underlying mechanism of HFpEF is left ventricle (LV) diastolic dysfunction (DD), which is also the potential mechanism underlying the most common arrhythmia, atrial fibrillation (AF) [7, 8]. Furthermore, DD is associated with increase in all-cause mortality and is not unique only to HFpEF, it is observed in patients with HF with reduced EF (HFrEF) as well [6].

The diagnosis of HFpEF, according to The European Society of Cardiology guidelines, is based on signs and symptoms of HF in patients with preserved LVEF of a non-dilated LV and elevated LV filling pressures with evidence of DD: impaired LV relaxation or increased LV diastolic stiffness [4, 9]. Invasive catheterization can provide the accurate diagnosis of DD in HFpEF. However, common practice is to establish the diagnosis non-invasively by echocardiographic assessment to ascertain preserved LV systolic function and estimate diastolic pressures [4, 6, 10]. Current DD echocardiographic recommendations [11] mostly focus on diagnostic criteria for patients in sinus rhythm, whereas DD diagnosis in AF is addressed as “in special populations”, pointing out that maximal left atria (LA) volume may be directly related to AF, and that Doppler assessment of LV diastolic function is limited by the variability in cycle length [11]. A multitude of parameters are presented that “can be used to predict LV filling pressures”, most of which are not consistently acquired in routine clinical studies.

LA maximal volume is part of the assessment of DD by echocardiography. Recently, LA phasic volumes have been suggested as an LA function assessment in relation to DD [12–14], since LA emptying actually reflects LV filling. Patients in sinus rhythm and patients with AF both have maximal and minimal LA volumes. Thus, LA global phasic function parameters can be compared and correlated to heart failure symptoms in patients in sinus rhythm and AF, where the active phase of LA contraction is absent. Moreover, as both LA and LV volumes are analyzed in tandem in the evaluation of LA function, the effects of variable cycle lengths in AF are probably minimized.

Myocardial mechanics, using feature tracking software, offer a unique method to elucidate both LV and LA function, creating strain and volume curves for analysis [15–17]. The objective of this study was to identify common LA function correlates of diastolic dysfunction and HF symptoms in patients in sinus and AF to suggest a method to diagnose DD in patients with AF and correlate them to HF symptoms in patients with preserved EF.

## Methods

### Patients

Medically stable patients between 19 and 90 years of age with history of AF (current or past) who had a transthoracic echocardiography assessment at Baruch Padeh Poriya Medical Center between January 2014 and October 2015 were retrospectively screened for this study. Patients were included if they had a LVEF ≥ 45%, and none of the following: more than mild valvular disease, cardiomyopathy, history of myocardial infarction or other non-cardiac sources of dyspnea, technically inadequate echocardiographic images, tachycardia, complete atrioventricular (AV) block, or electronic pacing. Patient had no prior diagnosis of HFpEF. After screening the departmental database, 109 patients met our inclusion criteria and remained after exclusion. These were divided into two groups according to their cardiac rhythm: patients with AF at the time of echocardiography and patients in sinus rhythm. Patients in sinus rhythm were then divided into two groups according to their diastolic function: patients with normal diastolic function (ND) and patients with abnormal diastolic function (DD). The control group was composed of 67 healthy subjects with no comorbidities and normal echocardiographic studies.

Patients were suspected to have HFpEF according to the following criteria of presenting symptoms: dyspnea, fatigue, weakness, reduced ability to exercise, pulmonary edema, and/or peripheral edema.

### Echocardiography

Routine clinical 2D and Doppler echocardiography were performed and collected retrospectively according to the American Society of Echocardiography recommendations. DD was determined for patients in sinus rhythm according to recent guidelines [11, 18, 19] and as previously published by our group. Diastolic function was evaluated using the mitral inflow pulsed-wave Doppler, septal and lateral mitral annular tissue-Doppler velocities, and pulmonary vein pulse wave Doppler velocities [5, 7]. DD was determined either as a pseudonormal (mitral E/A 0.8–1.9, E deceleration time (EDt) 140–280 ms) or restrictive pattern (E/A N 2, EDt b 140 ms). Evidence of elevated left atrial pressure (either E/E′ ≥ 14 or pulmonary S/D b 1.0) and left atrial (LA) enlargement was a pre-requisite for all patients (LA systolic diameter ≥ 38 mm for women, ≥ 40 mm for men) [20]. Since the guidelines refer only to specific parameters when EF is reduced to suggest (rather than diagnose) DD in patients in atrial fibrillation [11], LA emptying parameters were addressed to find correlates of DD and symptomatic HF.

**Fig. 1:**
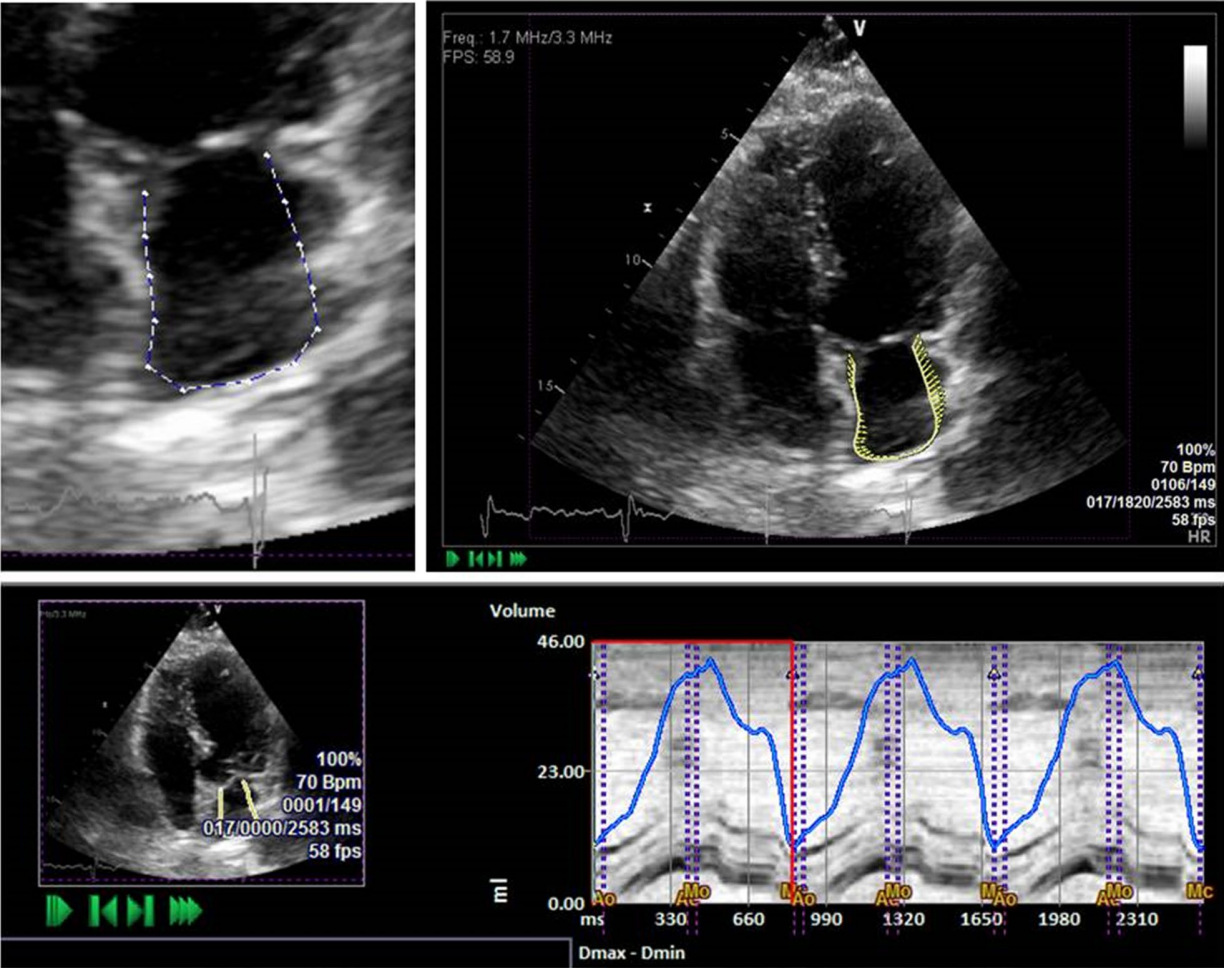
An example of measurements obtained by endocardial surface manual tracing using a point and click approach in a patient with sinus rhythm with diastolic dysfunction. The volume acquired automatically with endocardial tracking performed by the VVI software thereafter

**Fig. 2:**
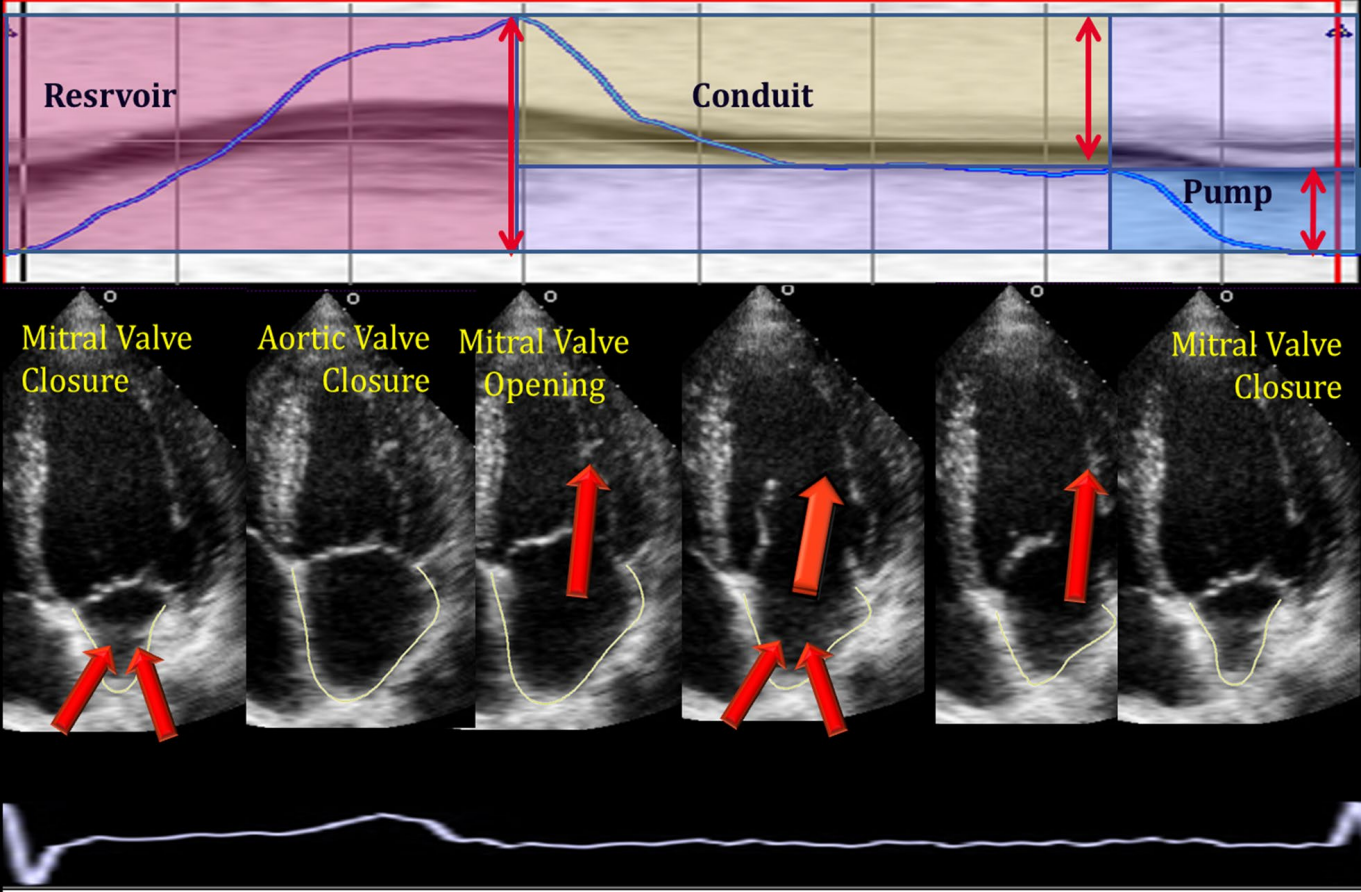
Left Atrial volumes and function, and the cardiac cycle acquired automatically by the VVI software following endocardial surface manual tracing using a point and click approach as seen in a patient with sinus rhythm on echocardiography

Myocardial mechanics were analyzed retrospectively. LA and LV measurements were performed offline by a single operator who was blinded to clinical and echocardiographic findings, using dedicated software (eSie VVI, us v.3.0.1.45 b.140211, Siemens Medical System, Mountain View). By using the dedicated clip editor, two to three cardiac cycles were selected for each representative view and the onset of R wave was used as the reference point for both LA and LV strain and volume curves [12, 13, 15, 16, 21]. Endocardial surface was manually traced using a point and click approach (Fig. 1) and then after automatically processed by the software. Apical views (4, 2 and 3 chambers) were analyzed for LV and LA Myocardial mechanics. Previous studies report consistently the fact that LV systolic and diastolic functions are tightly coupled. Additionally, association between LV systolic and diastolic strain together with LA strain and LV diastolic function was reported in studies using STE [11]. Variables included: strain (% shortening); global longitudinal strain (average strain at aortic valve closure, GLS); Global mechanical synchrony index (GMSi, equals to GLS/average of segmental strain peaks; equals to1 if peaks coincide at aortic valve closure); and the ratio of early diastolic strain rate to systolic strain rate (SR E/S ratio). Bi-plane (4 and 2 chambers) LV End diastolic and systolic volumes (ml) were assessed by VVI, as were LA myocardial mechanics, [11] LA reservoir strain, LA volumes (as seen in Fig. 2): LA maximal volume indexed to body surface area (Vmax-I, The volume measured just before the opening of the mitral valve, coinciding with the end systole phase of the LV on the echocardiography (ECG) trace); LA minimum volume indexed to body surface area (Vmin-I, The volume measured at the closure of the mitral valve, coinciding with the end diastole phase of the LV on the ECG trace); total emptying volume indexed to body surface area (, equals to Vmax-I minus Vmin-I); Conduit volume indexed to body surface area, (equals to LV stroke volume indexed minus LA total emptying volume indexed); and LA reservoir strain (%).

### Statistical analysis

Statistical analysis was performed using MedCalc® (version 13.1.2.0, Belgium). Data are presented as mean ± standard deviations (SD). Comparisons between groups’ variables were performed using the analysis of variance (ANOVA), followed by Tukey post-hoc analyses. Receiver operating characteristic (ROC) analysis and curve were used with a stepwise approach to determine models, calculate Odds Ratio (OR), 95% Confidence Intervals (CI) and the areas under the curve (AUC). Youden index was used in order to obtain the sensitivity and specificity of the correlation between variables in the different models and defined classification variables. Pairwise comparison was used in the multivariate statistical analysis between different models. Uni- and multivariable associates of DD and heart failure symptoms were assessed by logistic regression analysis. Statistical significance was defined using a *p* value of less than 0.05.

## Results

### Clinical characteristics

As summarized in Table 1, compared to the normal diastolic function with sinus rhythm patients (ND group) or normal controls, DD patients in sinus rhythm and patients with AF were more than a decade older. Furthermore, the AF group had more hypertension and dyspnea, whereas the DD group did not. In comparison with the ND group, the DD group had more diabetes mellitus (DM) and chronic kidney disease (CKD), whereas the AF group did not.

**Table 1:** Clinical characteristics

	Control n = 67	ND n = 32	DD n = 35	AF n = 42	*p* value
Age	51 ± 12	57 ± 16	73 ± 9*^,^**	73 ± 14*^,^**	0.004
Male, n (%)	33 (50)	19 (58)	9(25)	17 (38)	0.025
BMI (kg/m^2^)	29 ± 3	29 ± 4	30 ± 5	30 ± 7	NS
Diabetes Mellitus, n (%)	0 (0)	4 (12)*	14 (39)*^,^**	11 (24)*	< 0.001
Hypertension, n (%)	0 (0)	14 (42)*	23 (64)*	32 (71)*^,^**	< 0.001
Hyperlipidemia, n (%)	0 (0)	11 (33)*	15 (42)*	19 (42)*	< 0.001
Smoking, n (%)	0 (0)	6 (18)*	5 (14)	3 (7)	< 0.001
Renal Failure, n (%)	0 (0)	0 (0)	4 (11)*^,^**	2 (4)	< 0.001
Dyspnea, n (%)	0 (0)	1 (3)	8 (22)*	14 (31)*^,^**	< 0.001

*ND* Normal diastolic function; *DD* diastolic dysfunction; *AF* atrial fibrillation; * significant difference with the control group; ** significant difference with the Sinus ND group

#### Conventional 2-D Doppler echocardiographic characteristics

As summarized in Table 2, bi-plane LVEF was in the normal range (63% ± 4) and similar for all groups. Patients with DD and patients with AF demonstrated a higher calculated LV mass index, suggesting concentric LV hypertrophy. Pulmonary pressure showed gradual increments from normal controls through AF, ranging from normal values in the control group and the sinus ND group, to mildly elevated in patients with DD, and moderate pulmonary hypertension in the AF group.

**Table 2:** 2D Doppler echocardiographic characteristics

	Control, n = 67	ND, n = 32	DD, n = 35	AF, n = 42	*p* value
Heart rate (BPM)	65 ± 10	70 ± 15	67 ± 12	78 ± 22*^,^†	< 0.001
LVEDD (mm)	47 ± 4	50 ± 3	50 ± 3	50 ± 5	NS
LV mass index (g/m^2^)	70 ± 11	94 ± 19*	112 ± 23*^,^**	106 ± 27*	< 0.001
Ejection fraction, bi-plane (%)	66 ± 5	64 ± 4	64 ± 4*	61 ± 9	NS
Flow parameters					
Mitral E (cm/s)	75 ± 19	68 ± 13	84 ± 22**	–	0.01
Mitral E/A	1.2 ± 0.4	1 ± 0.4	1.2 ± 0.6	–	0.01
Mitral E/E’	8 ± 3	8 ± 2	12 ± 3*^,^**	–	0.004
Pulmonary pressure (mmHg)	26 ± 4	29 ± 5	36 ± 12*^,^**	43 ± 12*^,^**^,^†	< 0.001

*ND* Normal diastolic function; *DD* diastolic dysfunction; *AF* atrial fibrillation; * significant difference with the control group; ** significant difference with the Sinus ND group; † significant difference with the Sinus DD group; LVEDD, LV end diastolic diameter

### Left ventricular myocardial mechanics

As summarized in Table 3, Bi-plane LV ejection fraction calculated by speckle tracking of endocardial contour and longitudinal strain were decreased in groups of patients in sinus rhythm with history of AF and was lowest in patients with AF at examination. GMSi was significantly lower in patients with current AF, representing LV micro-dyssynchrony in this group. The ratio of early diastolic to systolic strain rate (SR E/S) was significantly increased in patients in AF in comparison to all other groups.

**Table 3:** The left ventricular myocardial mechanics

	Control, n = 67	ND, n = 32	DD, n = 35	AF, n = 42	*p* value
Ejection fraction (%)	61 ± 5	55 ± 7*^,^†	55 ± 9*	48 ± 8*^,^**^,^†	0.008
EDV (ml/m^2^)	64 ± 11	61 ± 13	65 ± 17	54 ± 19*^,^†	0.028
ESV (ml/m^2^)	24 ± 6	27 ± 8	30 ± 11	29 ± 14*	0.04
Strain (% shortening)					
Global longitudinal (GLS)	− 19 ± 2	− 19 ± 3	− 19 ± 4	− 14 ± 4*^,^**^,^†	< 0.001
Average of peaks (APS)	− 20 ± 2	− 20 ± 3	− 20 ± 4	-15 ± 4*^,^**^,^†	< 0.001
GMSi (GLS/APS) (%)	98 ± 2	95 ± 4*	93 ± 6*	91 ± 6*^,^**^,^†	< 0.001
SR E/S ratio	1.03 ± 0.14	1.08 ± 0.12	1.03 ± 0.23	1.2 ± 0.15*^,^**^,^†	0.031

*ND* Normal diastolic function; *DD* diastolic dysfunction; *AF* atrial fibrillation; * significant difference with the control group; ** significant difference with the Sinus ND group; † significant difference with the Sinus DD group; *EDV* end diastolic volume; *ESV* end systolic volume; *GLS* global longitudinal strain; *GMSi* global mechanical synchrony index; SR E/S ratio, The ratio of early diastolic strain rate to systolic strain rate

### Left atrial phasic volumes and myocardial mechanics

As summarized in Table 4, the volumes progressively increased from normal controls to ND group, DD group and the AF group, being the largest in patients in AF. The largest differences were noted in LA minimal volume index (Vmin-I). Functional parameters, such as the reservoir strain and diastolic emptying index (LA “ejection fraction”) were gradually decreased from normal controls to ND group, DD group and the AF group; passive emptying rate gradually decreased from normal controls to ND group and the DD group. Conduit volume remained with no significant difference among ND group, DD group and the AF group.

**Table 4:** The left atrium structure, function and strain characteristics

	Control, n = 67	ND, n = 32	DD, n = 35	AF, n = 42	*p* value
Indexed volumes (ml/m^2^)					
Vmax-I	32 ± 9	32 ± 10	44 ± 13*^,^**	51 ± 17*^,^**^,^†	< 0.001
Vmin-I	9 ± 4	12 ± 7	21 ± 10*^,^**	36 ± 17*^,^**^,^†	< 0.001
Total emptying volume i	24 ± 7	20 ± 6	23 ± 6*^,^**	15 ± 6*^,^**^,^†	< 0.001
Passive volume i	16 ± 6	11 ± 6*	10 ± 6*	12 ± 6*	< 0.001
Conduit volume i	15 ± 8	14 ± 6	13 ± 6	11 ± 6*	0.028
LA reservoir strain (%)	44 ± 11	49 ± 22	33 ± 19*^,^**	15 ± 8**^,^**^,^†	< 0.001
Diastolic emptying index (%) -(LA ejection fraction)	73 ± 9	65 ± 13*	54 ± 14*^,^**	33 ± 16*^,^**^,^†	0.003
Passive emptying duration (% cycle length)	30 ± 6	35 ± 11	31 ± 17	–	NS
Passive emptying rate (ml/%cycle length)	1.0 ± 0.3	0.6 ± 0.3*	0.5 ± 0.4*	–	< 0.001

*ND* Normal diastolic function; *DD* diastolic dysfunction; *AF* atrial fibrillation; * significant difference with the control group; ** significant difference with the Sinus ND group; † significant difference with the Sinus DD group; *Vmax-I* LA maximal volume indexed; *Vmin-I* LA minimal volume indexed

### Association with DD (patients in sinus rhythm)

**Fig. 3:**
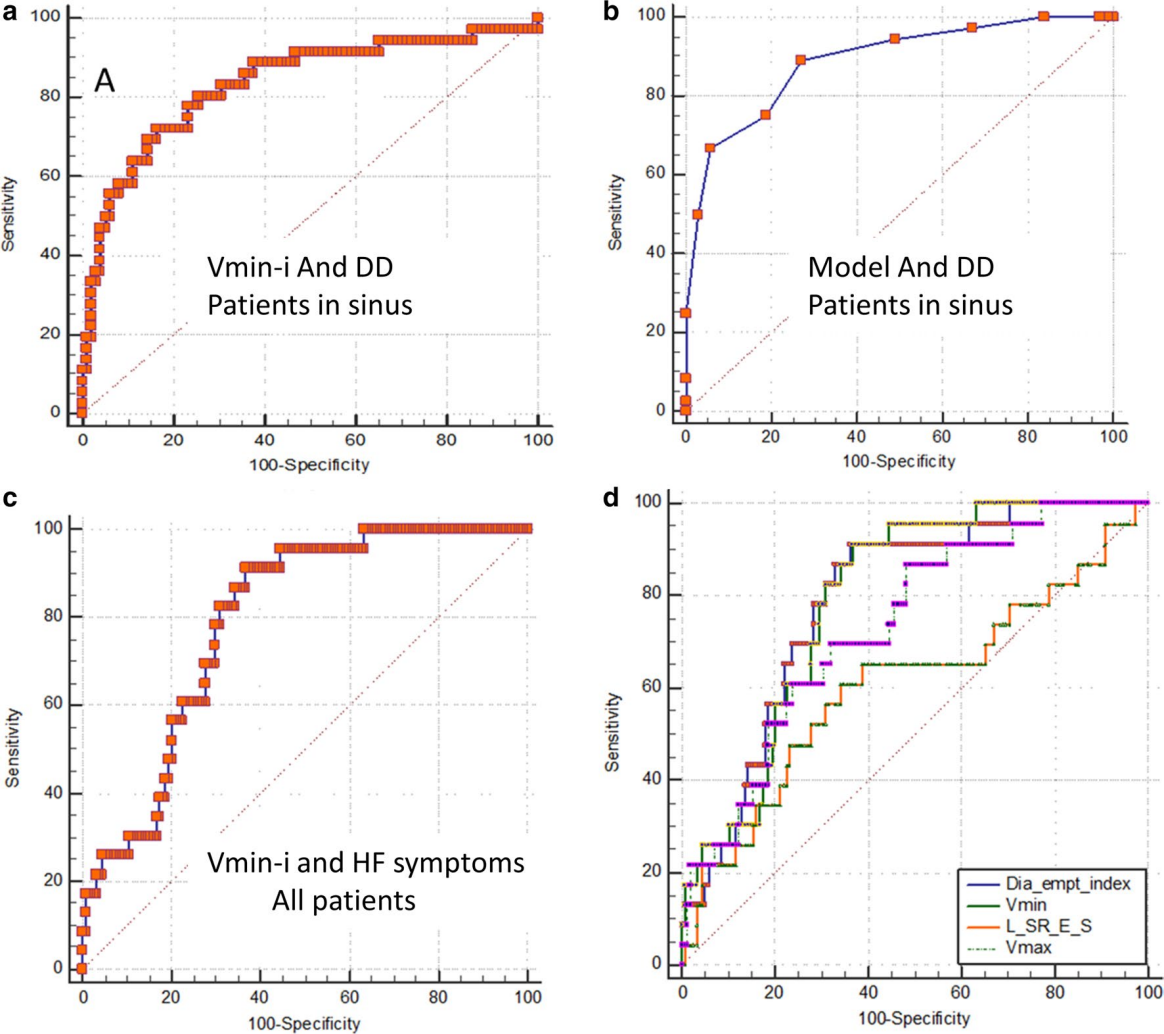
Receiver operating curves for association with DD and Dyspnea. *Vmin-I* LA minimal Volume index; *Vmax-I* LA maximal Volume index; *Dia-empt-index* Diastolic emptying index, (Vmax-I-Vmin-I)/Vmax-I; L-SR-E/S, longitudinal strain rate E to S ratio. **a** The association of LA minimal volume index (Vmin-I) with diastolic dysfunction in patients in sinus rhythm. **b** The association of age and Vmin-I model (coefficients derived from logistic regression) with diastolic dysfunction. **c** The association of Vmin-I with dyspnea/heart failure symptoms. **d** Comparison of various parameters for the association of heart failure symptoms

In order not to over-fit the model due to the small number of subjects and prevalence of DD, we needed to limit the number of variables in the model. At the first run we include age, LV functional parameters, and LA size and functional parameters. Age (HR = 1.12, CI 1.06–1.18, *p* < 0.0001) and Vmin-I (HR = 1.2, CI = 1.08–1.27, *p* < 0.0001) were the only associates of DD in a stepwise logistic regression analysis for all subjects in sinus rhythm (parameters rejected were: LVEF, LA total emptying volume, LA reservoir strain, and diastolic emptying index).. Re-running the model with other LV functional parameters, such as LV GLS, the ratio of early diastolic to systolic strain rate or pulmonary pressure replacing LVEF in the model, did not change the results, as they were all rejected. The ROCs for the association of DD to Vmin-I ≤ 16 ml/m^2^ alone vs. Vmin-I + age model were not different (AUC 83%) (Fig. 3a, b).

### Association with heart failure symptoms (all patients)

Applying the same model to all patients with heart failure symptoms yielded Vmin-I as the single significant parameter associated with heart failure symptoms (HR = 1.04, CI 1.02–1.05, *p* < 0.0001) (Fig. 3c). Comparing ROCs for various parameters (Table 5, Fig. 3d) shows overlapping curves for Vmin-I and LA diastolic emptying index, both better and significantly different from the LV strain Rate E/S ratio and LA Vmax-I.

**Table 5:** ROC pairwise comparisons (*p* values) for prediction of dyspnea/heart failure symptoms

Variable	AUC	Diastolic emptying index	Vmin-I	SR E/S ratio	Vmax-I
Diastolic emptying index	0.778	1	0.5	0.01	0.1
Vmin-I	0.762	0.5	1	0.02	0.03
SR E/S ratio	0.594	0.01	0.01	1	0.02
Vmax-I	0.694	0.1	0.03	0.02	1

*Vmim-I* LA minimal volume indexed; SR E/S ratio, The ratio of early diastolic strain rate to systolic strain rate; *Vmax-I* LA maximal volume indexed

Creating a new association parameter—patient in sinus, with normal diastolic function (controls and patients with history of AF) vs. the rest of the study patients (patients in sinus rhythm with history of AF with diastolic dysfunction + patients in AF), Vmin-I remained as the single associate with heart failure symptoms, rejecting all other parameters in a stepwise logistic regression model. The hazard ratio (HR) of the latter group for heart failure was found to be HR = 38 (CI 5–293, *p* < 0.0001) with an AUC of 0.8, sensitivity of 63%, and specificity 96%.

## Discussion

To the best of our knowledge, this is the first study that aims to find common LV/LA myocardial mechanics parameters to demonstrate DD, using STE in patients with AF.

Few studies are related to the subject of atrial dysfunction in circumstances of AF in patients with HFpEF. For example, our study may seem complementary to the work of Reddy et al. [22] which demonstrated the mechanical decline of LA function, resulting in AF, in patients with HFpEF. While the above mentioned study aimed to determine the consequence of LA dysfunction to emphasize the AF burden in HFpEF patients, the goal of our study was to find common LV/LA myocardial mechanics parameters, to demonstrate DD in patients with AF and associate these parameters with symptomatic HFpEF.

Patients with AF at the echocardiography examination and patients in sinus rhythm with DD were clinically and echocardiographically similar. In fact, DD augments the risk of developing AF and is probably the underlying mechanism for AF [7, 8, 23]. The high clinical and echocardiographic resemblance between AF and sinus-DD groups suggests a high rate of DD in patients with AF.

As seen in previous studies [24–26], Bi-plane LV ejection fraction calculated by speckle tracking of endocardial contour was decreased relatively to conventional 2-D Doppler echocardiographic assessment, due to the higher precision of determining end-systole and diastole together with defining the endocardial boundaries.

In patients in sinus rhythm, E/E’ and Vmin-I were significantly correlated with DD, while end systolic LA volume indexed (Vmax-I) was not. Notably, although E/E’ can be assessed in AF, mitral E velocities and annular E’ tissue velocities are not measured simultaneously and probably require averaging in multiple beats to bear significance [16]. Left atrial volumes (maximal, minimal, conduit and reservoir function) are measurable in AF and sinus rhythm. LA emptying directly represents LV filling, and is especially and probably less rate dependent, as both LA emptying and LV filling are measured in in the same cycle. Furthermore, in multiple-variable analysis, the left atrial minimal volume was correlated with both DD and presence of dyspnea/heart failure symptoms, indicating that the minimal volume of the atria during LV diastole and heart failure symptoms are likely related. Since the presence of DD cannot be accurately determined in the AF group, as no gold-standard is available, the study results suggest correlation of the minimal volume of the LA with symptoms of heart failure and raised filling pressures.

Patients presented with sinus rhythm and DD or AF had more symptoms of heart failure (HR = 38, CI 5–293, *p* < 0.0001) than patients presented with normal diastolic function and in sinus rhythm (controls and patients with history of AF). This high specificity that we found suggests that heart failure diagnosis could likely be excluded in patients in sinus rhythm and normal diastolic function with history of AF. In a multivariable model, Vmin-I was correlated with dyspnea more than any other parameters, including Vmax-I and SR E/S ratio which was previously found to correlate with LV end diastolic pressure [16]. Left atrial strain has been shown to aid in the categorization of DD [27]. The advantage of measuring the left atrial minimal volume is that it is simple to perform as a single volume measurement that can be derived from a conventional echocardiography assessment without the need of any software post-processing. In addition to association of LA with DD and HF symptoms, pulmonary hypertension is also presented by dyspnea and is a common complication of left HF. Pulmonary hypertension is also regarded as a component of the DD score, according to the latest guidelines [11]. In our study, pulmonary pressure was demonstrated to be significantly correlated with Vmin-I and may contribute to the mechanism of DD causing HF.

Katbeh, et al. [28] presented the diagnostic advantage of LA strain to differ between HFpEF and non-cardiac causes of dyspnea in patients with paroxysmal AF. As shown, our above results distinguish the probability of the existence of DD in AF patients, by means of measuring the minimal volume of the left atrium, a rhythm independent variable in order to suggest the presence of diastolic dysfunction in patients with suspected HFpEF, regardless of the heart rhythm they present during their examination, especially and notably, AF.

As previously published, Vmin correlates better to DD than Vmax [29]. Atrial volume is correlated to atrial fibrillation [30], The left atrial volume is affected by the various phases of the heart cycle and therefore it depends upon LV and LA systolic function, LV stiffness (reduced compliance) and geometry. All of these parameters define how the LA is passively filled, passively and actively emptied, and also how it remodels in size and stiffness. Stiffening and reduced compliance of the LA and LV can lead to an enlargement of the LA [12, 15], and thus may preserve the pressure gradients needed to maintain diastolic filling. LA size may also exceed the optimal sarcomere length of the LA myocytes and consequently reduces contractility. Diastolic function can be defined by not only the conventional Vmax-I, which reflects the size, but also by LA Vmin-I, which expresses both size and function. As we showed, Vmin-I was tightly related to the total LA diastolic emptying (LAEF). Therefore, high Vmin-I may indicate DD, which can be assessed regardless of cardiac rhythm, as an important tool for its estimation both in sinus rhythm and AF. As it is also associated with HF symptoms, it probably bears more than just a designation of DD and is likely a more comprehensive clinical informative parameter. Further studies are needed to assess its prognostic power as well.

### The clinical significance of the study

Vmin demonstrated a strong correlation to dyspnea/HF and is thus proposed, as a simple, single, rhythm-independent variable that could be used to ascertain the presence of diastolic dysfunction in patients suspected of heavy diastolic dysfunction, regardless of their heart rhythm they have during their examination. The suggested measurement can be assessed at the bedside, as an efficient test that does not require extensive off-line post-processing tools and is usually already acquired in standard echocardiographic studies. The use of Vmin in standard echocardiographic studies could facilitate in reflecting a much more realistic quantification of incidence and prevalence of HFpEF, and thus may help identify patients at different risks of developing dyspnea and HF. If the right diagnosis can be made, progress to better treatment strategies may be found and thereafter could be offered to patients, with an impact that would perhaps reduce hospitalizations, the economic burden on the society, and may be eventually augment a better quality of life for patients as well. Secondly, based on our findings we could assume that a patient referred for an echocardiographic examination, following the history of AF, found in sinus rhythm with ND at the time of examination, has probably a very low risk of developing dyspnea or HF symptoms.

The left atrial minimal volume was significantly correlated with both diastolic dysfunction and symptomatic heart failure. Thus, the left atrial minimal volume may be used as a rhythm independent variable to suggest the presence of diastolic dysfunction in patients with suspected HFpEF, regardless of the heart rhythm they have during their examination. In addition to the presence of HF symptoms, we found that a Vmin-I cut off > 16 ml/m^2^, in the presence of sinus rhythm or AF, may suggest increased probability of HFpEF that might need a closer follow-up and earlier therapeutic intervention. Measuring Vmin-I is a simple, single, rhythm independent, bed-side, non-time consuming echocardiographic assessment, which does not require extensive off-line post processing tools and can be incorporated easily in a routine standard echocardiographic examination [13–15, 31, 32].

### Limitations

The study was conducted retrospectively in a tertiary health care center. Selection bias cannot be excluded. Most patients were examined in an outpatient setting during a single visit, and therefore, medical data such as laboratory results (e.g. NT-proBNP) and follow-up assessments were unavailable. In addition, gold-standard measurements, such as cardiac catheterization, were also not available in this ambulatory setting. Offline analysis of echocardiographic exams was done by a single operator. Some echocardiographic studies were excluded due to inadequate echocardiographic quality for off-line strain analysis 3D strain and volume were not used, and this is due to the intention of applying methods used in real-life clinics. Excluded patients’ demographic and clinical characteristics did not differ from included patients. Naturally, patients with AF were much older and composed of a majority of women compared to the studied control group, composed of relatively young and healthy subjects, and therefore were not age nor sex matched. Furthermore, we acknowledge patient’s comorbidities could affect LA function and DD, and yet due to small size of groups we could not match groups by these different risk factors.

## Conclusions

The left atrial minimal volume may be used to suggest the presence of DD in patients with AF and help with the identification of patients at high risk for developing HFpEF. The use of Vmin-I should be validated by larger multicenter studies. Furthermore, patients with a history of AF who undergo echocardiographic examination in sinus rhythm and demonstrate normal diastolic function at the time of examination may be at lower risk for developing HFpEF.

## Data Availability

The data is available from the corresponding author upon request.

## Abbreviations

DD: Diastolic dysfunction
HFpEF: Heart failure with preserved ejection fraction
HFrEF: Heart failure with reduced ejection fraction
AF: Atrial fibrillation
ND: Normal diastolic function
HF: Heart failure
LV: Left ventricle
LA: Left atria
LVEF: Left ventricle ejection fraction
AV: Atrioventricular
EDt: E deceleration time
GLS: Global longitudinal strain
GMSi: Global mechanical synchrony index
SR E/S ratio: Systolic strain rate
ECG: Echocardiography
SD: Standard deviations
HR: Hazard ratio
ANOVA: Analysis of variance
OR: Odds ratio
CI: Confidence intervals
AUC: Areas under the curve
DM: Diabetes mellitus
CKD: Chronic kidney disease
